# Sex-Specific Cell Types and Molecular Pathways Indicate Fibro-Calcific Aortic Valve Stenosis

**DOI:** 10.3389/fimmu.2022.747714

**Published:** 2022-02-24

**Authors:** Veronika A. Myasoedova, Ilaria Massaiu, Donato Moschetta, Mattia Chiesa, Paola Songia, Vincenza Valerio, Valentina Alfieri, Romain Capoulade, Daniela Trabattoni, Daniele Andreini, Elvira Mass, Valentina Parisi, Paolo Poggio

**Affiliations:** ^1^ Centro Cardiologico Monzino, Istituto di Ricovero e Cura a Carattere Scientifico (IRCCS), Milan, Italy; ^2^ Developmental Biology of the Immune System, Life and Medical Sciences (LIMES) Institute, University of Bonn, Bonn, Germany; ^3^ Dipartimento di Scienze Farmacologiche e Biomolecolari, Università degli Studi di Milano, Milano, Italy; ^4^ Department of Electronics, Information and Biomedical Engineering, Politecnico di Milano, Milan, Italy; ^5^ Dipartimento di Medicina Clinica e Chirurgia, Università degli Studi di Napoli Federico II, Napoli, Italy; ^6^ L’institut du thorax, INSERM, CNRS, University of Nantes, CHU Nantes, Nantes, France; ^7^ Department of Biomedical and Clinical Sciences “Luigi Sacco”, University of Milan, Milan, Italy; ^8^ Dipartimento di Scienze Mediche traslazionali, Università degli Studi di Napoli Federico II, Napoli, Italy

**Keywords:** aortic valve stenoses, fibrosis, inflammation, immune system, sex-difference

## Abstract

**Background:**

Aortic stenosis (AS) is the most common valve disorder characterized by fibro-calcific remodeling of leaflets. Recent evidence indicated that there is a sex-related difference in AS development and progression. Fibrotic remodeling is peculiar in women’s aortic valves, while men’s leaflets are more calcified. Our study aimed to assess aortic valve fibrosis (AVF) in a severe AS cohort using non-invasive diagnostic tools and determine whether sex-specific pathological pathways and cell types are associated with severe AS.

**Materials and Methods:**

We have included 28 men and 28 women matched for age with severe AS who underwent echocardiography and cardiac contrast-enhanced computed tomography (CT) before intervention. The calcium and fibrosis volumes were assessed and quantified using the ImageJ thresholding method, indexed calcium and fibrosis volume were calculated by dividing the volume by the aortic annular area. For a deeper understanding of molecular mechanisms characterizing AS disorder, differentially expressed genes and functional inferences between women and men’s aortic valves were carried out on a publicly available microarray-based gene expression dataset (GSE102249). Cell types enrichment analysis in stenotic aortic valve tissues was used to reconstruct the sex-specific cellular composition of stenotic aortic valves.

**Results:**

In agreement with the literature, our CT quantifications showed that women had significantly lower aortic valve calcium content compared to men, while fibrotic tissue composition was significantly higher in women than men. The expression profiles of human stenotic aortic valves confirm sex-dependent processes. Pro-fibrotic processes were prevalent in women, while pro-inflammatory ones, linked to the immune response system, were enhanced in men. Cell-type enrichment analysis showed that mesenchymal cells were over-represented in AS valves of women, whereas signatures for monocytes, macrophages, T and B cells were enriched men ones.

**Conclusions:**

Our data provide the basis that the fibro-calcific process of the aortic valve is sex-specific, both at gene expression and cell type level. The quantification of aortic valve fibrosis by CT could make it possible to perform population-based studies and non-invasive assessment of novel therapies to reduce or halt sex-related calcific aortic valve stenosis (CAVS) progression, acting in an optimal window of opportunity early in the course of the disease.

## Introduction

Aortic stenosis (AS) is the most common valve disorder characterized by fibro-calcific remodeling of valve leaflets (1). Progressive aortic valve calcification (AVC) occurs in both sexes and multiple pieces of evidence indicate a sex-related difference in aortic valve composition in AS (2, 3). Cardiac computed tomography (CT) is recognized as a high-quality technique for AVC evaluation. To date, sex-specific CT thresholds of AVC have been implemented in clinical practice since it is now recognized that women have less AVC burden than men (4, 5).

Recently, Voisine et al. (6) using aortic valve histology, confirmed that women have more fibrotic remodeling compared to men. In addition, it has been shown that the quantitative assessment of both calcific and non-calcific (*i.e.*, fibrotic) aortic valve tissue by contrast-enhanced CT correlated well with AS severity (7). In addition, this study showed that, even if the indexed non-calcific volume is slightly lower in women compared to men, the fibro-calcific ratio is significantly higher in women. However, the analysis was performed in patients with mild, moderate, and severe AS, and only 4 women were classified with severe AS.

Furthermore, profound differences in mineral composition and morphology between sexes were recently reported, suggesting that aortic valve calcification follows different mineralization pathways in men and women (8). Of note, recent *in vitro* studies highlighted that human valve interstitial cells (VICs) isolated from men had a significantly higher calcification potential under pro-osteogenic and pro-inflammatory stimuli than cells isolated from women (9, 10). However, no study has identified sex-specific pathways and/or cell types linked to severe AS.

Therefore, we sought i) to confirm whether aortic valve fibrosis (AVF) is a more important contributor to AS in women compared to men, implementing a severe AS cohort, and ii) to determine which sex-specific pathological pathways and cell types are associated with severe AS. Moreover, with the aim to explore the global involvement in AS of circulating inflammatory cells, interstitial and endothelial cells, and the different cell subtypes contributing to the onset of pathology, we focused our study on the whole tissue.

## Materials and Methods

### Study Population

Eighty consecutive patients (48 men and 32 women) who underwent Doppler echocardiography and cardiac CT within 3 months before aortic valve surgery were enrolled in Centro Cardiologico Monzino IRCCS (2018-2019). Exclusion criteria were AS due to rheumatic disease or radiotherapy, previous infective endocarditis, previous severe adverse reactions to an iodinated contrast agent, body mass index (BMI) ≥38 kg/m^2^, and severely impaired renal function (estimated glomerular filtration rate < 30 ml/min/1.73 m^2^). In the final analysis, 56 age and sex-matched patients, with both bicuspid (BAV) and tricuspid (TAV) aortic valve morphology, were included. This study was approved by the Institutional Review Board of Centro Cardiologico Monzino IRCCS (n. R1348/20-CCM1418), and the participants gave written informed consent. The study protocol conforms to the ethical guidelines of the 1975 Declaration of Helsinki.

### CT Examination and Imaging Acquisition

**Figure 1:**
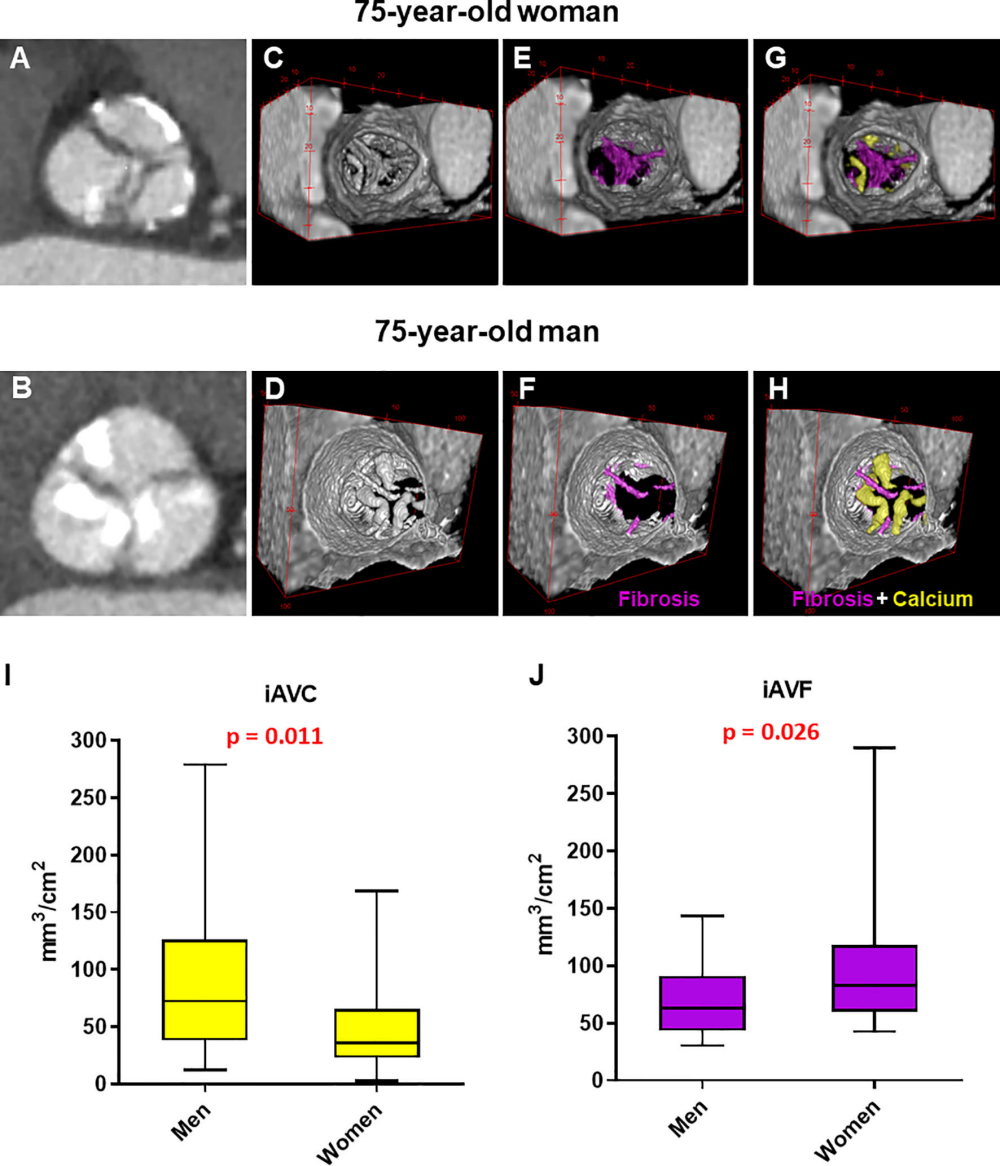
Manual analysis of aortic valve calcium and fibrosis by contrast-enhanced computed tomography in men and women. **(A, B)** Computed tomography images reoriented into en face aortic valve view from a woman and a man with severe aortic stenosis. **(C–H)** Representative 3D contrast-enhanced volume-rendered views of aortic valve from a woman and a man with fibrotic tissue enhanced in violet and calcium in yellow. Box plots showing the difference in indexed aortic valve calcium **(I)** and fibrosis **(J)** volume between men (n = 28) and women (n = 28) with severe AS. Unpaired non-parametric Mann-Witney test was used to evaluate differences.

Cardiac CT (Revolution CT, GE Healthcare, WI) was performed using the following parameters: section configuration 256x0.625 mm, voxel size 0.625 mm, spatial resolution along the X-Y planes 0.23 mm, gantry rotation time 280 msec, and iterative reconstruction. A BMI-adapted scanning protocol (tube voltage and current) was used (11). All patients received a 50-mL bolus of contrast (Iomeprol 400 mg/mL, Bracco, Milan, Italy). Images were post-processed using dedicated software for AVC assessment (3Mensio Valves™, 3Mensio Medical Imaging, Maastricht, The Netherlands) (12) with manual correction. Contrast attenuation values (Hounsfield Units, HU) and contrast-to-noise ratio were measured at the level of the ascending aorta. Total AVF was assessed based on HU ranging between 30 and 350, adjusting the upper threshold by increments of 25 HU in either direction until blood pool was not highlighted, as previously published for non-calcific aortic valve tissue evaluation (2, 7). The calcium and fibrosis volumes were assessed and quantified using the ImageJ (Java-based image processing program developed at the National Institutes of Health) thresholding method (**Figures 1A–H**) (13). Indexed contrast-enhanced CT calcium volume and fibrosis volume were calculated by dividing the volume by the aortic annular area for each patient.

### Deep Learning Workflow

The workflow diagram of the deep learning analysis is reported in **Supplementary Figure S1**. Each CT scan is composed of 256 or 224 slices (512 x 512 pixels) and stored in a DICOM object. In order to remove useless anatomical structures, such as aorta or ventricles, we selected the consecutive slices containing the aortic valve, which allows decreasing the computational burden and improving the overall network performances. Each selected slice was then used to perform a manual segmentation in order to generate the ‘ground truth’ images.

Thirty-eight out of 56 randomly selected samples were then used to train a U-Net model, with a default parameter, to segment the aortic valve. The remaining 20 samples served as the test set. The U-Net model was implemented with default hyperparameters, using the ‘Keras’ package; concerning the training step, we set the ‘Root Mean Squared (RMS) propagation’ optimizer to minimize the ‘binary cross-entropy (BCE) dice’ loss function, the number of epochs = 50 and the batch size = 10. The Dice Score was used to assess the segmentation accuracy, comparing the predicted mask and the ground truth.

Finally, the predicted masks were combined with the corresponding starting frames in order to extract the pixel intensity (Hounsfield Unit) of the aortic valve and, then, calculate the fibrosis and calcium volumes. The Pearson’s correlation index (r_p_) allowed assessing the consistency between the automatic predictions and the calculations of fibrosis and calcium volumes, made by human operators.

### Gene Expression Data Processing

Gene expression data were retrieved from Gene Expression Omnibus (GEO) public repository [GEO accession number: GSE102249 (14)]; this dataset collects the transcriptomic analysis, performed by microarray technology, of 240 valve tissues, coming from 120 males and 120 females. We re-annotated the Illumina probe ID, using the Ensembl human gene annotation (GRCh38, version 97); then we removed outliers and normalized data by log2 transformation and quantile normalization. The analysis was performed by R (v. 3.6.3).

### Pathways’ Enrichment Analysis

Gene set enrichment analysis (GSEA) was conducted using GSEA pre-ranked tool (v 4.0.3) (15) and the biological functions were inferred by taking advantage of gene ontology biological processes (GO-BP), Reactome, WikiPathways, and BioCarta databases. To reduce redundancy and visually interpret GSEA results, the most significant GO-BP/Reactome/BioCarta pathway gene sets were identified by a false discovery rate (FDR) < 0.1. The network was drawn through the Enrichment Map software v.3.3.2 (16) implemented as a plug-in in Cytoscape v.3.8.2 (17).

### Cell-Type Enrichment Analysis

Cellular composition of stenotic aortic valve tissue in women and men was inferred by the xCell R package (18), starting from the gene expression dataset of 240 samples. xCell raw scores were generated and transformed applying the spill-over compensation. Then, we assessed the association between the xCell scores and sex by Wilcoxon rank-sum test. The log_2_ fold-change (logFC) of each cell type scores between men and women was computed and the pValues were adjusted with FDR correction. It is important to note that xCell results are expressed in terms of enrichment scores. Therefore, any inference on the cell counts or proportion of cell types is not provided.

### Statistical Analysis

The data were analyzed using IBM SPSS statistic 26 software. Continuous variables were expressed as mean ± standard deviation (SD) or, when variables had a skewed distribution, as median and interquartile range, while categorical ones were reported as frequency and percentage. Between-group differences were evaluated by Student t-test and by Pearson Chi-square (χ2) test. A pValue < 0.05 was considered to be statistically significant. Unpaired non-parametric Mann-Witney test was used to evaluate differences in indexed aortic calcification (iAVC) and fibrosis (iAVF).

Differential expression analysis between male and female samples was performed through the limma R/Bioconductor package (19). This statistical analysis was performed in the R environment v3.6.3. We deemed genes as significantly different at an FDR-adjusted pValue < 0.05. The robustness of the differential expression analysis results was assessed by exploring the histogram of the pValue distribution.

## Results

### Contrast CT Evaluation of iAVC and iAVF

We studied 56 patients (28 men and 28 women) matched for age (71 ± 11 years) with severe AS (mean gradient 47.4 ± 13.6 mmHg, aortic valve area 0.82 ± 0.23 cm^2^, and ejection fraction 64 ± 7%). **Table 1** summarizes the patients’ characteristics. In brief, there was no difference between men and women in major cardiovascular risk factors, valve phenotype (17 BAV and 39 TAV in total), nor pharmacological treatment. As expected, men had higher body surface area and annulus diameter than women (1.89 ± 0.14 *vs*. 1.67 ± 0.17 m^2^, respectively; p < 0.001) and (36.1 ± 3.0 *vs*. 31.9 ± 2.8 mm, respectively; p < 0.001), while women had lower aortic valve area than men (AVA; 0.74 ± 0.2 *vs*. 0.91 ± 0.2 cm^2^, respectively; pValue = 0.007) but similar indexed AVA (0.44 ± 0.15 *vs.* 0.48 ± 0.12 cm^2^/m^2^, respectively; pValue = 0.262).

**Table 1:** Characteristics of sex and age-matched aortic stenosis (AS) patients that underwent contrast-enhanced cardiac computed tomography.

Variables	Men	Women	Total
	n = 28	n = 28	pValue
**Age, years**	71.4 ± 10.8	71.5 ± 11.1	0.961
**Diabetes, n (%)**	7 (25)	5 (18)	0.515
**Hypertension, n (%)**	22 (79)	21 (75)	0.752
**Dyslipidaemia, n (%)**	22 (79)	16 (57)	0.086
**Smoking, n (%)**	9 (32)	8 (27)	0.771
**Body mass index, kg/m^2^**	26.9 ± 3.8	26.2 ± 6.0	0.588
**eGFR, mL/min/1.73m^2^**	72.3 ± 17.6	80.1 ± 16.3	0.053
**BSA, m^2^**	1.89 ± 0.14	1.67 ± 0.17	**<0.001**
**LVEF, n (%)**	64.0 ± 7.8	64.5 ± 7.2	0.798
**Peak aortic jet velocity, m/s**	4.4 ± 0.6	4.4 ± 0.7	0.916
**Mean gradient (mmHg)**	46.7 ± 11.8	48.0 ± 15.2	0.726
**Aortic valve area, cm^2^**	0.91 ± 0.21	0.74 ± 0.23	**0.007**
**Aortic valve area indexed (cm^2^/m^2^)**	0.48 ± 0.12	0.45 ± 0.15	0.365
**Annulus diameter, mm**	36.1 ± 3.0	31.9 ± 2.8	**<0.001**
**Antiplatelet agents, n (%)**	17 (61)	13 (46)	0.284
**ACE -inhibitors, n (%)**	8 (27)	11 (39)	0.397
**Angiotensin Receptor Blockers, n (%)**	10 (36)	7 (25)	0.383
**Beta-blockers, n (%)**	17 (61)	18 (64)	0.783
**Calcium channel blockers, n (%)**	6 (21)	7 (25)	0.752
**Diuretics, n (%)**	11 (39)	7 (25)	0.252
**Anti-diabetic drugs, n (%)**	4 (14)	4 (14)	1.000
**Lipid-lowering drugs, n (%)**	18 (64)	11 (39)	0.061

ACE, angiotensin converting enzyme; eGFR, estimated glomerular filtration rate; LVEF, left ventricular ejection fraction. pValue < 0.05 are reported in bold.

Our results confirmed that, for a same hemodynamic severity in both sex, women had significantly lower iAVC compared to men (36 [95% Confidence Interval (CI): 27-49] *vs.* 72 [95% CI: 48-114] mm^3^/cm^2^, respectively; pValue = 0.011). Furthermore, we showed that, in severe AS patients, the fibrotic tissue composition (*i.e.*, iAVF) of the valve leaflets was significantly higher in women compared to men (83 [95% CI: 65-111] *vs.* 63 [95% CI: 45-82] mm^3^/cm^2^, respectively; pValue = 0.026; **Figures 1I, J**). Finally, the fibro-calcific ratio, which indicates the predominance of valve fibrosis if > 1.0, was significantly higher in women compared to men (2.57 [95% CI: 1.16-3.62] *vs.* 0.78 [95% CI: 0.43-1.95], respectively; pValue = 0.0018). Interestingly, the significant difference in iAVF volume between men in women were observed only in TAV morphology (**Supplementary Figure S2**).

**Figure 2:**
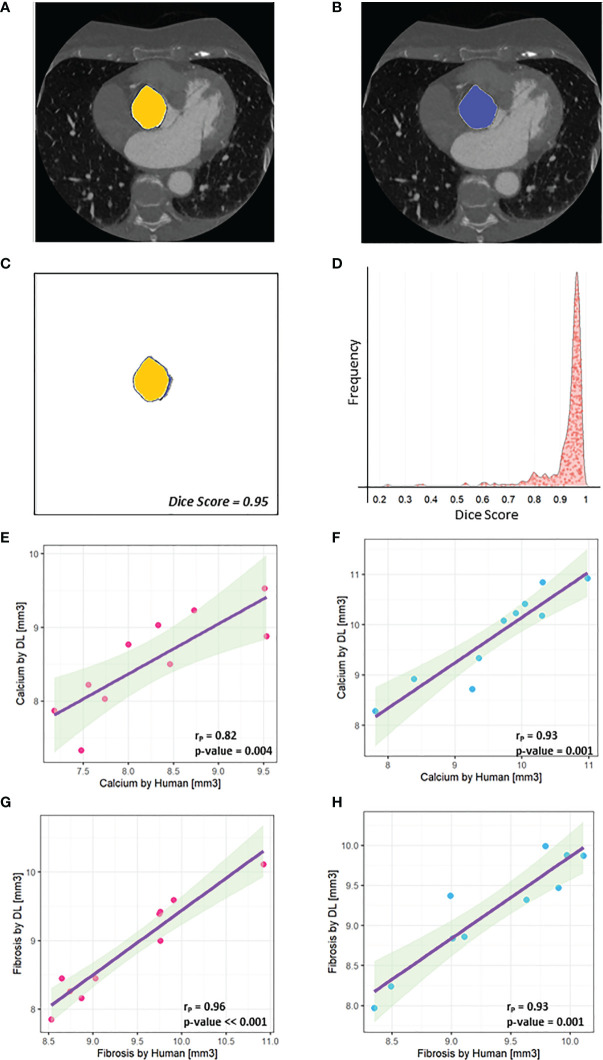
U-net performances. **(A)** An example of mask designed by the human operator (‘Ground truth’) is highlighted in yellow and combined with the original frame. **(B)** An example of mask predicted by U-Net is highlighted in blue and combined with the original frame. **(C)** The high overlapping level between A and B is assessed by the Dice Score (0.95). **(D)** Distribution of Dice Scores shows that a higher level of overlapping (Dice score = 0.93 +- 0.09) was obtained between the learned U-Net model and the human operator. **(E)** A Pearson’s correlation index rP = 0.82 was gained comparing the calcium volume in 10 female patients calculated by human (x-axis) and obtained by the deep learning automatic framework on the test set. **(F)** A Pearson’s correlation index rP = 0.93 was gained comparing the calcium volume in 10 male patients calculated by human (x-axis) and obtained by the deep learning automatic framework on the test set. **(G)** A Pearson’s correlation index rP = 0.96 was gained comparing the fibrosis volume in 10 female patients calculated by human (x-axis) and obtained by the deep learning automatic framework on the test. **(H)** A Pearson’s correlation index rP = 0.93 was gained comparing the fibrosis volume in 10 male patients calculated by human (x-axis) and obtained by the deep learning automatic framework on the test set.

As the identification of valve in CT scans is a crucial step and could depend on the human operator experience, we validated our findings training a deep learning model (U-Net) to automatically recognize valve area in each frame without prior knowledge and calculate calcium and fibrosis volumes (**Figures 2A–C**). As shown in **Figure 2D**, we observed a high level of overlapping (Dice Score = 0.93+-0.09) between 400 predictions of valve area, performed by a human operator and the U-Net. Moreover, a Pearson’s correlation index r_p_ = 0.82 in 10 females and r_p_ = 0.93 in 10 males was gained comparing the calcium volume calculated by human (x-axis) and obtained by the deep learning automatic framework on the test set (i.e., the dataset containing images never seen by U-net during training), while a r_p_ = 0.96 in 10 females and r_p_ = 0.93 in 10 males was gained comparing the fibrosis volume calculated by human (x-axis) and obtained by the deep learning automatic framework on the test set (**Figures 2E–H**).

### Stenotic Aortic Valve Sex-Specific Gene Expression

The public microarray-based gene expression dataset of 240 human stenotic aortic valves was analyzed to identify the genes and the biological pathways differentially expressed between women and men with severe AS.

The sex classification for each sample was performed based on the expression levels of XY-linked genes. In particular, Ribosomal Protein S4 Y-Linked 1 (RPS4Y1) and X Inactive Specific Transcript (XIST) genes were considered sex-specific markers, to split 120 male and 120 female samples. During the differential expression analysis, the XY-linked genes and pseudogenes were filtered out.

**Figure 3:**
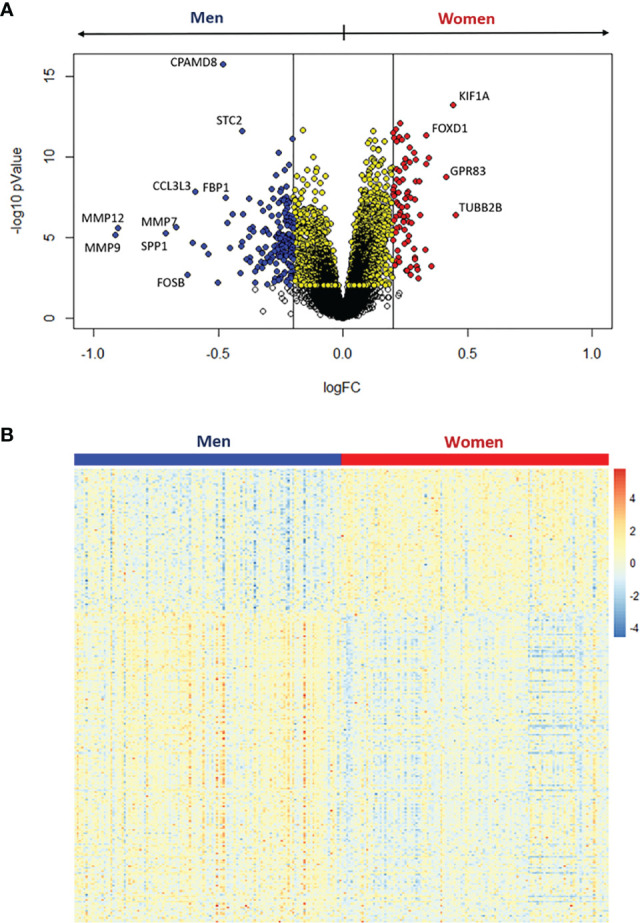
Differential expression analysis between women and men stenotic aortic valves. **(A)** Volcano plot of the differentially expressed genes. Yellow dots represent significant differentially expressed genes at adjusted pValue < 0.05, whereas red and blue dots represent upregulated genes in women and men, respectively, with both the adjusted pValue < 0.05 and the absolute log2 fold change (logFC) > 0.2. **(B)** The heatmap shows the expression levels for the significantly upregulated genes with logFC > 0.2 in women (red) and men (blue).

We observed that 3105 genes were significantly regulated between samples of women and men (**Figure 3**). Setting a threshold of 0.2 log_2_ fold-change (logFC), 80 genes were up-regulated in women, while 173 genes were up-regulated in men (**Figure 3A** and **Supplementary Figure S3**). The heatmap (**Figure 3B**) shows that these 253 differentially expressed genes separate the specimens by sex. The top ten up-regulated genes in women and men are listed in **Tables 2** and **3**, respectively.

**Table 2:** Top 10 upregulated autosomal genes in women.

TOP 10 UPREGULATED GENES IN WOMEN			
Symbol	Annotation	logFC	Adjusted pValue
TUBB2B	Tubulin Beta 2B Class	0.45	2.31 e-5
KIF1A	kinesin family member 1A	0.44	4.37 e-10
GPR83	G Protein-Coupled Receptor 72/83	0.41	4.27 e-7
SLN	Sarcolipin	0.35	6.31 e-3
CHST6	Carbohydrate Sulfotransferase 6	0.34	3.13 e-8
FOXD1	Forkhead Box D1	0.33	7.81 e-9
DPT	Dermatopontin	0.33	1.10 e-7
COL6A6	Collagen Type VI Alpha 6 Chain	0.33	1.07 e-3
SOX8	SRY-Box Transcription Factor 8	0.31	2.49 e-5
RASL11B	RAS Like Family 11 Member B	0.31	6.68 e-7

**Table 3:** Top 10 upregulated autosomal genes in men.

TOP10 UPREGULATED GENES IN MEN			
Symbol	Annotation	logFC	Adjusted pValue
MMP9	Matrix metallopeptidase 9	-0.91	2.03 e-4
MMP12	Matrix metallopeptidase 12	-0.90	9.40 e-5
SPP1	Secreted phosphoprotein 1	-0.71	1.62 e-4
MMP7	Matrix metallopeptidase 7	-0,67	8.99 e-5
FOSB	FosB Proto-Oncogene, AP-1 Transcription Factor Subunit	-0,62	1.66 e-2
S100A8	S100 Calcium Binding Protein A8	-0.60	4.81 e-4
CCL3L3	Chemokine (C-C motif) ligand 3	-0.59	2.29 e-6
AQP9	Aquaporin 9	-0.56	6.88 e-4
HMOX1	Heme oxygenase (decycling) 1	-0.54	1.58 e-3
FOS	Fos Proto-Oncogene, AP-1 Transcription Factor Subunit	-0.50	3.86 e-2

### Functional Inferences Form Gene Expression Analysis of Women and Men Stenotic Aortic Valves

**Figure 4:**
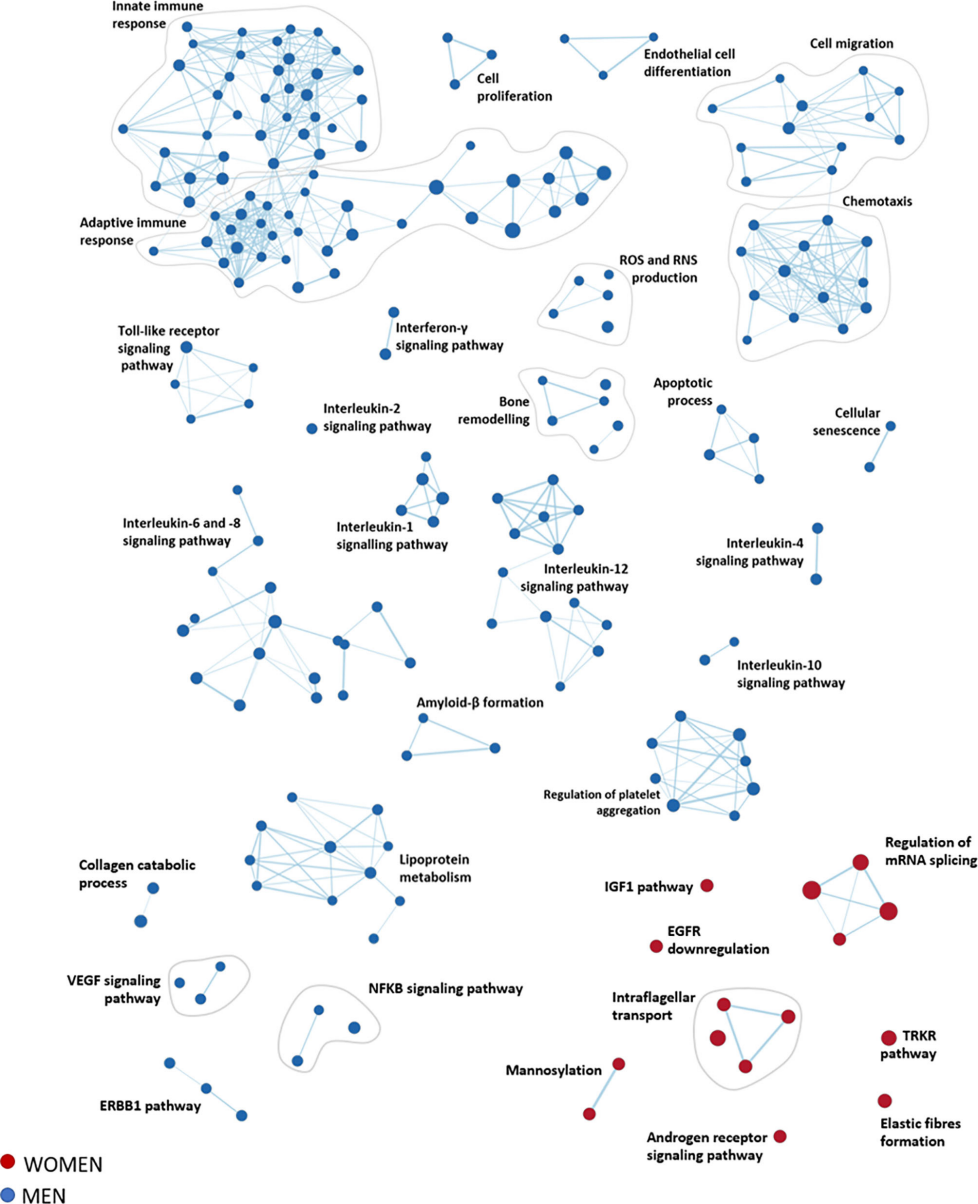
Functional inferences from gene expression analysis of women and men stenotic aortic valves. The enrichment network shows the pathway sets (nodes) that are associated (FDR qValue < 0.1) with women (red) and men (blue), where the node size is proportional to the gene-set size.

A total of 4979 pathways characterizing women and men’s aortic valves were identified using GSEA (**Supplementary Tables S1**, **S2**). To enhance the interpretation of the results, we clustered the most significant pathways (FDR qValue < 0.1) into 33 macro-pathways (8 related to women and 25 related to men). The obtained enrichment network was drawn (**Figure 4**), considering the computed NES and the gene counts (**Supplementary Figure S4**).

The most representative pathways associated with women were related to the regulation of mRNA splicing, intraflagellar transport, post-translational modification (*i.e.*, mannosylation), elastin fibers formation, insulin-like growth factor 1 pathway, and regulation of tyrosine kinase receptor.

In contrast, the most representative pathways associated with men belonged to the immune system response (innate and adaptive immunity), cell migration, and chemotaxis, constituting the wider groups of highly interconnected gene sets. Other molecular pathways enriched in men are inflammation (interleukin 1, 2, 4, 6, 8, 10, and 12), bone remodeling, cell proliferation, endothelial cell differentiation, cellular senescence, lipoprotein metabolism, reactive oxygen species, and reactive nitrogen species production, and regulation of platelet aggregation.

### Enrichment Analysis of Sex-Specific Cell Types in Stenotic Aortic Valve Tissues

To reconstruct the cellular composition of the stenotic aortic valve from bulk transcriptome, we employed the xCell method to convert the gene expression profile to enrichment scores of several immune and stromal cell types across specimens (18).

Aortic valve leaflets harbor a heterogeneous cell population (20). Thus, to deconvolute cell types within the bulk transcriptional data, we selected 28 cell-type-specific gene expression profiles (**Supplementary Table S3**).

**Figure 5:**
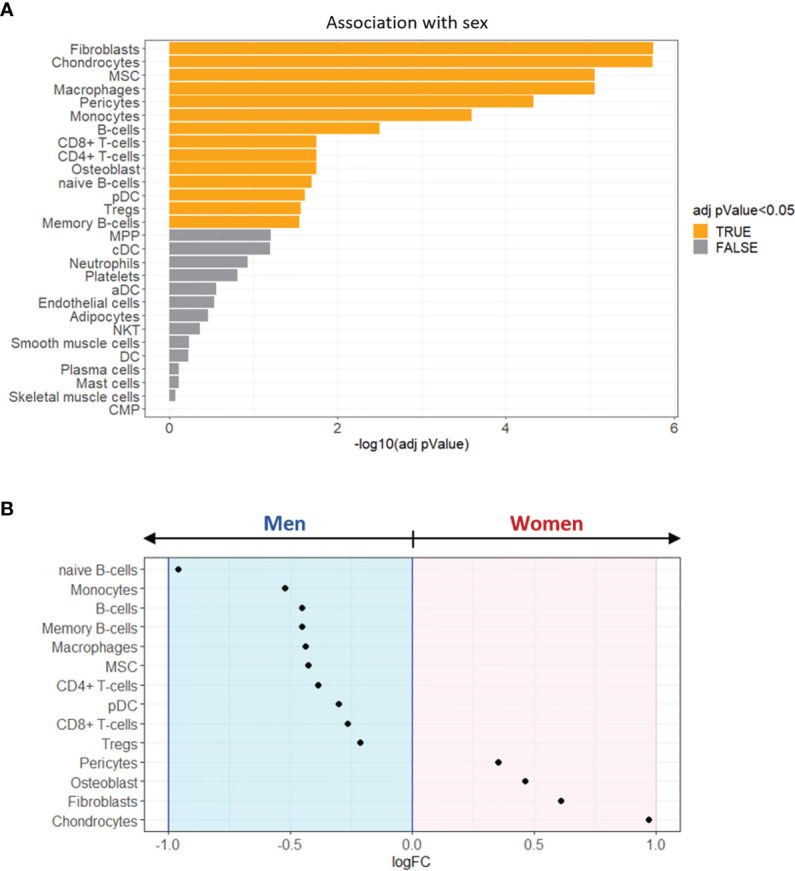
Enrichment analysis of sex-specific cell types in stenotic aortic valve tissues. **(A)** Bar graph showing the sex-related association of xCell scores, performed by Wilcoxon rank-sum test. The cell types with FDR adjusted pValue < 0.05 (orange) were considered significantly associated with one of the two sexes. **(B)** The dot plot shows the log2 fold change (logFC) of xCell scores, being associated with women (logFC > 0; pink background) or with men (logFC < 0; light blue background).

The cell-type enrichment analysis showed that 14 cell types significantly differed between women and men (adjusted pValue < 0.05, **Figure 5A**). Of note, as shown in **Figure 5B**, 4 cell types were identified as over-represented in women (logFC > 0) and 10 in men (logFC < 0).

The most representative cell types in women’s stenotic aortic valve leaflets were chondrocytes, fibroblasts, osteoblasts, and pericytes. Conversely, men’s stenotic aortic valve leaflets were characterized by the presence of i) innate immune system cells (monocytes, macrophages, and dendritic cells), ii) adaptive immune system cells (B and T lymphocytes), and iii) mesenchymal stem cells.

## Discussion

Our study demonstrated a sex dimorphism in the fibrotic content of severe AS evaluated by contrast-enhanced CT and the expression profile of human stenotic aortic valve specimens confirm sex-dependent processes. Pro-fibrotic processes were prevalent in women, while pro-inflammatory ones, linked to the immune response system, were enhanced in men. Cell-type enrichment analysis revealed that mesenchymal cells were over-represented in women’s AS valves, whereas transcriptional signatures for immune cells such as monocytes, macrophages, T and B cells were enriched in men ones.

CAVS is the most frequent valvular heart disease in the Western world. No medical therapy is currently available (21) even if multiple studies have been performed to elucidate the molecular and cellular mechanisms underlining the progression of this debilitating and fatal disease (22, 23).

Recently, it has been shown, both at the clinical and experimental level, that fibro-calcification processes occurring during AS development and progression could be different in women and men (24, 25). Indeed, Voisine et al. (6) revealed that women had less calcification and more fibrotic remodeling than men, evaluated by CT and histological analysis. Our data, obtained from severe AS patients, confirmed these findings and demonstrated the possibility to quantify the fibrotic tissue present in the aortic valve in a semi-automated fashion. Of note, a recent study evaluating individual valve tissue components in AS showed that adding non-calcific parameters (*e.g.*, fibrosis) to the AVC score improved diagnostic and prognostic accuracy (2). Therefore, the routine use of CT could allow monitoring the valve remodeling process from its early stages (*i.e.*, aortic valve sclerosis) that are primarily due to fibrotic processes. Finally, the quantification of aortic valve fibrosis by CT could make it possible to perform population-based studies and non-invasive assessment of novel therapies aimed at reducing or halting sex-related AS progression, acting in an optimal window of opportunity that may be early in the course of the disease (26).

To date, sex-specific molecular pathways and cell types involved in AS progression at the tissue level remain elusive. *In vitro* experiments performed on human VICs stimulated with interferon-α and lipopolysaccharide showed that the Janus kinases (JAK)/signal transducer and activator of transcription (STAT) signaling pathway plays an important role in fibro-calcific processes, leading to a significant increased calcification in male VICs (9). Furthermore, the same group highlighted that STAT-1 was a potent inducer of hypoxia-inducible factor-1α (HIF-1α), resulting in increased sex-related VICs calcification (male VICs > female VICs) (10). Of note, a gene expression study performed on healthy porcine VICs showed that there is a sex-related propensity for CAVS (27). Our analysis discovered more than 250 genes differentially expressed between women and men stenotic aortic valves, for the first-time revealing a complex sex-specific molecular network at the end-stage of CAVS. We have found that genes predominantly expressed in women’s stenotic aortic valves belong to pro-fibrotic pathways, while genes expressed in men belong to pro-inflammatory – mostly due to immune cell infiltrates – and pro-calcific ones. Remarkably, a recent study evaluating biomarkers of patients with AVC revealed that circulating proteins associated with fibrosis are mainly expressed in women, while, men had a positive association with biomarkers linked to inflammation and calcification (28).

CAVS is a chronic inflammatory disease involving innate and adaptive immune responses, characterized by extensive immune cell infiltration such as macrophages, dendritic cells, T cells, B cells, and mast cells (29). In addition, it has been shown that the crosstalk between these immune cell infiltrates and VICs plays a pivotal role in CAVS development and progression (29, 30). Surprisingly, up to 15% of cells present within healthy aortic valve leaflets are CD45^+^, a marker of the hematopoietic lineage, and their number increases substantially when CAVS specimens are considered (31). Nevertheless, whether the influence of immune cells, in the CAVS contest, is sex-specific is still unknown.

Our enrichment analysis of sex-specific cell types in stenotic aortic valve tissues underlines that, in women, the immune cells’ influence was mild compared to the contribution of chondrocytes, fibroblasts, osteoblasts, and pericytes. In contrast, the presence of macrophages, dendritic cells, B and T lymphocytes, in men’s stenotic aortic valves, could indicate a sex-specific involvement of the immune system in CAVS.

Our study has some potential limitations. In particular, the public gene expression levels of 240 AS patients (14) were not corrected for the probable confounding factors, such as age, hypertension, diabetes mellitus, kidney disease, and AS severity, because of the unavailability of additional information. Indeed, previous studies showed that women present higher NYHA class and greater prevalence of chronic kidney disease, while men present a higher prevalence of CAD, peripheral artery disease (PAD), and diabetes (32, 33). However, the molecular results obtained from the gene expression analysis permitted to confirm the MDCT inferences on a validated publicly available dataset. However, the implication of immune response in men and women should be deeply investigated. Finally, our study was focused on the end-stage of CAVS disease. Certainly, the inclusion of patients with early/mild stages of disease could allow deeper investigation related to molecular mechanisms underlying CAVS. Indeed, the results of our study suggested that routine use of non-invasive CT could allow monitoring valve remodeling progression from the earliest stage (*i.e.*, aortic valve sclerosis), which is mostly due to fibrotic processes. Finally, while there is evidence suggesting differences in pathology between men and women, a common pathological process manifesting clinically at different stages in men and women cannot be excluded.

## Conclusion

Calcific aortic valve stenosis is a complex disease with many known molecular and cellular processes involved and, currently, there is no therapeutic option besides aortic valve replacement.

Our data suggest that the fibro-calcific process of the aortic valve is sex-specific, both at gene expression and cell type level. Thus, we should focus on sex-specific processes when looking for new CAVS pharmacological targets and novel prognostic and diagnostic approaches. The quantification of aortic valve fibrosis by CT could make it possible to perform population-based studies and non-invasive assessment of novel therapies aimed at reducing or halting sex-related CAVS progression, acting in an optimal window of opportunity that may be early in the course of the disease.

## Data Availability Statement

The raw data supporting the conclusions of this article will be made available by the authors, without undue reservation.

## Ethics Statement

This study was approved by the Institutional Review Board of Centro Cardiologico Monzino IRCCS (n. R1348/20-CCM1418). The patients/participants provided their written informed consent to participate in this study.

## Author Contributions

The manuscript was mainly written by PP, VM, and IM with contributions from DM, MC, PS, VV, VA, RC, DT, DA, EM, and VP. VM, IM, and PP analyzed and interpreted data with contributions from MC, DT, and DA. VP and DT contributed to the design of the study. DM, MC, PS, VV, VA, RC, DT, DA, EM, and VP critically revised the manuscript. All authors reviewed and approved the manuscript.

## Funding

This work was supported by the Italian Ministry of Health funds (Ricerca Finalizzata: GR-2019-12370560 and GR-2018-12366423; ERA-CVD: PICASSO-JTC-2018-042) and by Fondazione Gigi e Pupa Ferrari ONLUS (FPF-14). EM was funded by the Deutsche Forschungsgemeinschaft (DFG, German Research Foundation) under Germany’s Excellence Strategy – EXC2151 – 390873048”. RC was supported by a “Connect Talent” research chair from Région Pays de la Loire and Nantes Métropole.

## Conflict of Interest

The authors declare that the research was conducted in the absence of any commercial or financial relationships that could be construed as a potential conflict of interest.

## Publisher’s Note

All claims expressed in this article are solely those of the authors and do not necessarily represent those of their affiliated organizations, or those of the publisher, the editors and the reviewers. Any product that may be evaluated in this article, or claim that may be made by its manufacturer, is not guaranteed or endorsed by the publisher.
