# Supplementary material for: Sex-Specific Cell Types and Molecular Pathways Indicate Fibro-Calcific Aortic Valve Stenosis
Source: Front Immunol. 2022 Feb 24;13:747714. doi: 10.3389/fimmu.2022.747714 (PMC8907138; doi:10.3389/fimmu.2022.747714)
Supplement: Supplementary file 4 [file Image_1.pdf]

SUPPLEMENTARY FIGURES

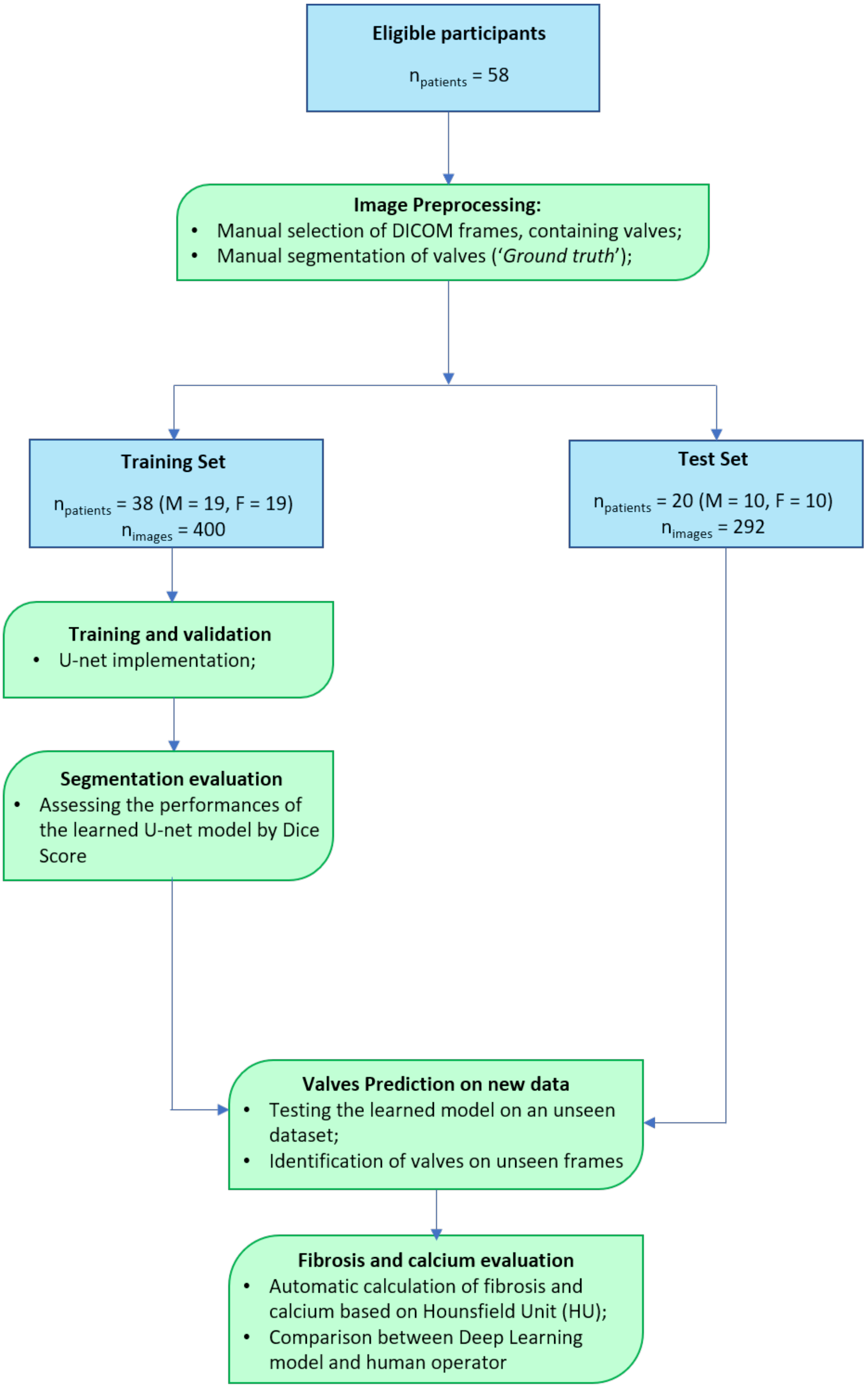

Supplementary Figure S1. Deep Learning Workflow for the aortic valve segmentation and the calculation of fibrosis and calcium volume. The block diagram allows describing the evolution of the dataset (light blue blocks) and the actions performed on each dataset (green blocks).

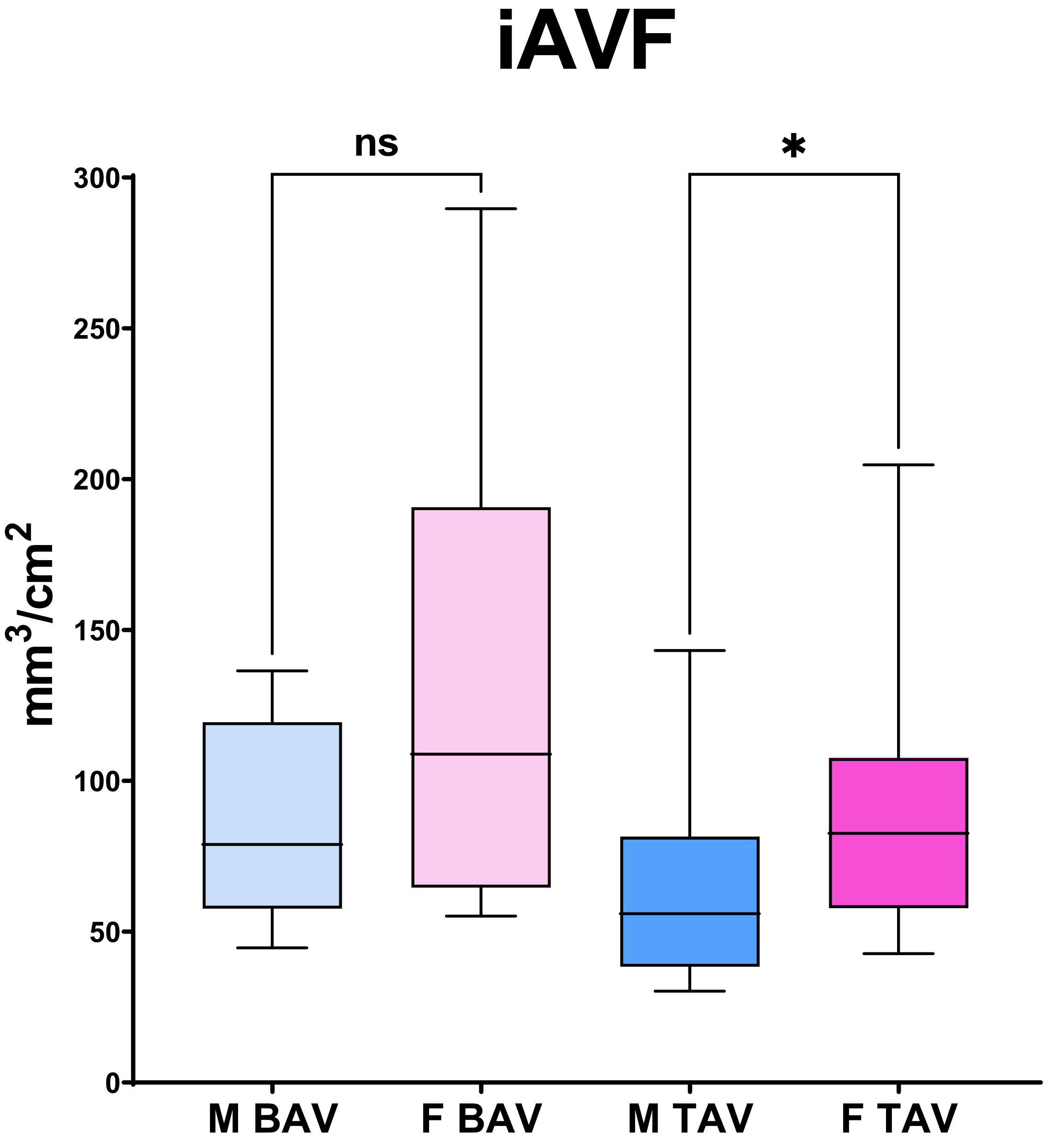

Supplementary Figure S2. Box plots showing the difference in indexed aortic valve fibrosis volume between men (n = 9) and women (n = 8) with severe aortic stenosis (AS) in bicuspid aortic valve (BAV) and between men (n = 19) and women (n = 20) with severe AS in tricuspid aortic valve (TAV). Unpaired non-parametric Mann-Witney test was used to evaluate differences. NS: non significative; *: significance p <0.05

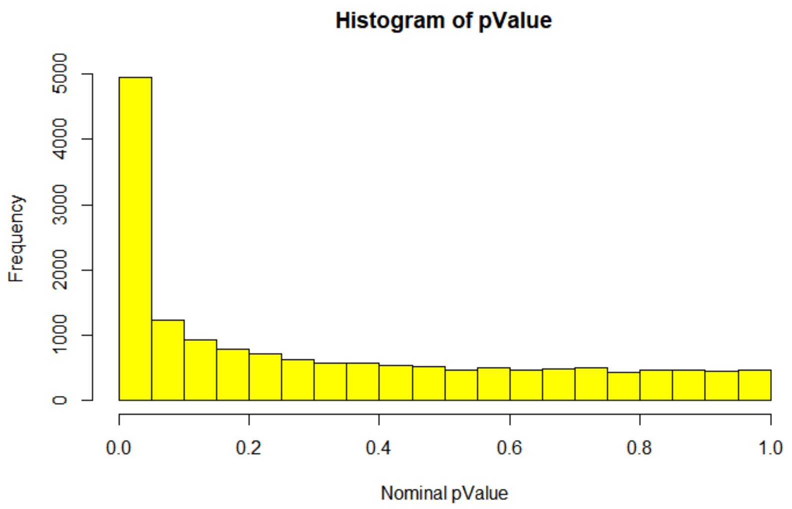

Supplementary Figure S3. Histogram of the pValue distribution. Differential analysis between women and men stenotic aortic valves.

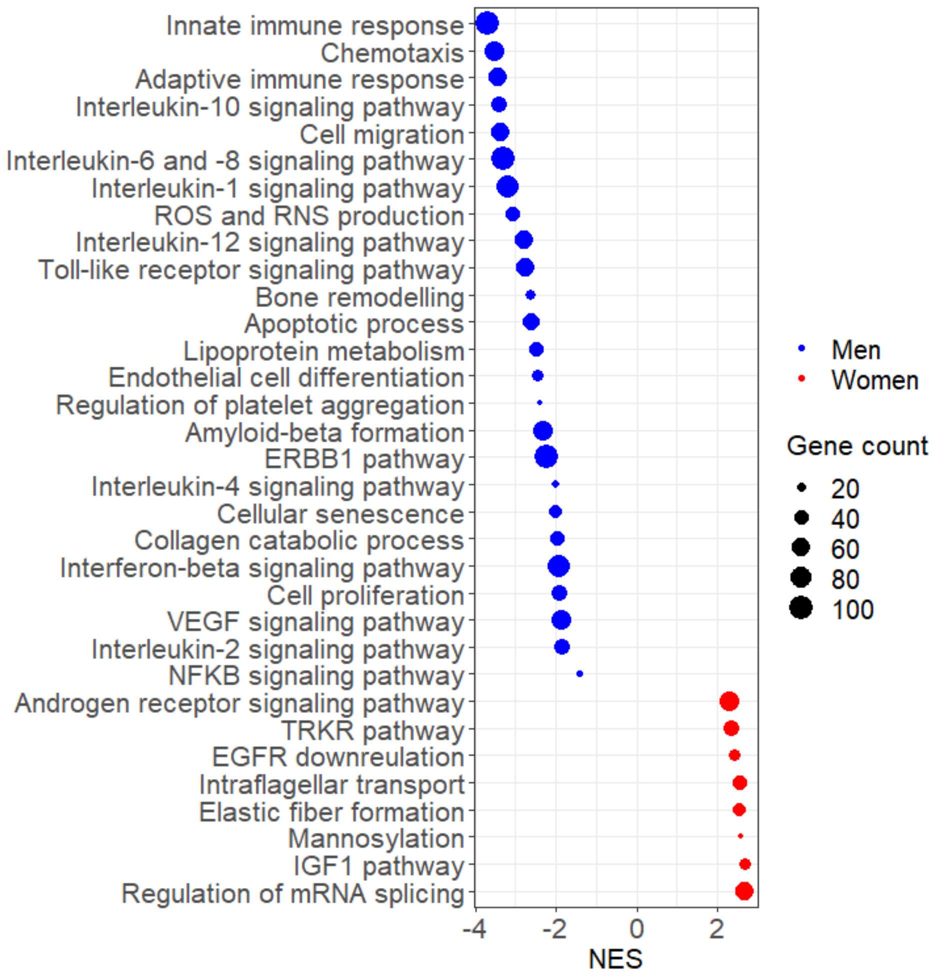

Supplementary Figure S4. Dot plot representing NES and gene count. Representative pathways (FDR qValue < 0.1) normalized enrichment score is reported for each pathway. The dot color refers to sex and the size is proportional to the gene-set size.
